# Investigations, management and outcome of neonates presenting with distal intestinal obstruction: challenging the need for contrast enemas

**DOI:** 10.1007/s00383-024-05725-w

**Published:** 2024-06-09

**Authors:** Hannah Wells, Georgina Bough, Francesca Stedman, Abiola Rachel Ekerin, Nigel J. Hall

**Affiliations:** 1 Department of Paediatric Surgery and Urology, Southampton Children’s Hospital, Southampton, UK; 2 University Surgery Unit, Faculty of Medicine, University of Southampton, Tremona Road, Southampton, SO16 6YD UK

**Keywords:** Neonatal distal intestinal obstruction, Hirschsprung disease, Meconium ileus, Meconium plug syndrome, Cystic fibrosis

## Abstract

**Purpose:**

To characterise the investigations, management and ultimate diagnosis of neonates with distal intestinal obstruction.

**Methods:**

Retrospective review of term (> 37 weeks) neonates with admission diagnosis of distal intestinal obstruction over 10 years (2012–2022). Patient pathways were identified and associations between presentations, response to treatments and outcome investigated.

**Results:**

A total of 124 neonates were identified and all included. Initial management was colonic irrigation in 108, contrast enema in 4, and laparotomy in 12. Of those responding to irrigations none underwent contrast enema. Ultimately, 22 neonates proceeded to laparotomy. Overall, 106 had a suction rectal biopsy and 41 had genetic testing for cystic fibrosis. Final diagnosis was Hirschsprung disease (HD) in 67, meconium ileus with cystic fibrosis (CF) in 9, meconium plug syndrome in 19 (including 3 with CF), intestinal atresia in 10 and no formal diagnosis in 17. Median length of neonatal unit stay was 11 days (7–19).

**Conclusions:**

Initial management of neonates with distal bowel obstruction should be colonic irrigation since this is therapeutic in the majority and significantly reduces the need for contrast enema. These infants should all have suction rectal biopsy to investigate for HD unless another diagnosis is evident. If a meconium plug is passed, testing for CF is recommended. Evaluation and therapy are multimodal and time consuming, placing burden on resources and families.

## Introduction

Distal intestinal obstruction in the neonatal period is a relatively common clinical presentation to the paediatric surgical team, typically presenting with two or more of the following symptoms: abdominal distension, vomiting and failure to pass meconium. Usually, these neonates present within the first few days of life. Standard teaching mandates thorough clinical examination, stabilisation and attempted decompression of the gastrointestinal tract if immediate surgical intervention is not warranted. Management thereafter involves consideration of underlying diagnosis followed by appropriate investigations, monitoring for signs of re-obstruction and subsequently definitive treatment where indicated. Traditional surgical texts and the existing literature typically discuss intestinal obstruction in general [1, 2] or the underlying pathology [3–6] and reflect back on initial presentation rather than considering epidemiology and management of neonates presenting with distal intestinal obstruction. As a result, there are a lack of data reporting management strategies, investigations, and outcomes of neonates with distal intestinal obstruction but in whom the underlying pathology is not yet known.

In addition, the precise indications for, and value of individual investigations to identify the cause of the distal intestinal obstruction are unclear. With a range of modalities on offer to the neonatal surgeon including plain abdominal radiograph, anal calibration, colonic irrigations, lower gastrointestinal contrast enema, suction rectal biopsy (SRB), cystic fibrosis genetic testing and laparotomy, greater clarity on the appropriate diagnostic pathway based on individual presenting features may allow for a more streamlined and efficient approach to the management of these cases. Finally, within the existing literature, there are a lack of data reporting final outcome of neonates presenting with the admission phenotype (working diagnosis) of distal intestinal obstruction. Rather, most reported series and contemporary texts focus on cohorts with a specific final diagnosis. A greater understanding of the outcome for babies based on their clinical presentation may enhance clinical decision making as well as inform parental counselling. Having a better understanding of the likelihood of a final diagnosis of for instance Hirschsprung disease, meconium ileus, intestinal atresia, meconium plug syndrome and importantly no pathology at all would likely be informative for the clinical team and may guide investigative strategy.

We, therefore, aimed to characterise the investigations, management and ultimate diagnosis of neonates presenting with distal intestinal obstruction to a tertiary neonatal surgical unit and to consider the value of the range of investigations and therapies in their diagnostic evaluation.

## Materials and methods

### Study population and demographics

All term (≥ 37 + 0 weeks gestation) neonates presenting to a single tertiary paediatric surgical neonatal unit between January 2012 and December 2022 with a clinical presentation of distal intestinal obstruction were identified from a prospectively maintained database. Whilst this presentation was based on overall clinical assessment, only neonates with at least 2 of abdominal distension, vomiting or no passage of meconium by 48 h from birth were included. Neonates born at < 37-weeks gestation and those with any form of anorectal malformation were excluded. Demographic, clinical and operative data were recorded from the medical records and data were managed and analysed in Microsoft Excel. Given the nature of the study and the data we anticipated, we planned to perform descriptive statistical analysis and also investigate associations between presenting clinical features, response to different treatment modalities and final outcome.

During the study period, all surgeons in our unit followed a consistent approach to the initial management of these neonates. So long there was no indication for immediate laparotomy, our standard approach is to aim to achieve gastrointestinal decompression with colonic irrigations initially. If adequate decompression could not be achieved by one or more irrigations then subsequent investigations and procedures including contrast enema, suction rectal biopsy (SRB), cystic fibrosis testing and laparotomy are guided by response to irrigations and subsequent course, but not protocolised.

Colonic irrigations are performed by passing an appropriately sized (e.g. 10CH in a term infant) soft catheter per rectum and instilling warmed 0.9% sodium chloride in 20 ml aliquots under gravity or by syringe gently if the catheter becomes obstructed with meconium. The effluent is allowed to drain through the catheter by gravity or leaks around it. The instillation is continued until the effluent obtained flows clear. There is not a strict maximal amount of fluid used for the colonic irrigation, so long as the fluid is returned rather than retained. The catheter is often advanced further during the procedure so long as there is no resistance, to achieve as adequate decompression as proximal as possible. This procedure usually takes around 20–30 min and care should be taken to maintain normothermia. If irrigation achieves only partial decompression, then so long as the infant is deemed stable enough by the treating clinician, the irrigation can be repeated a few hours later until adequate decompression is achieved. Following initial decompression by irrigation, colonic irrigations are typically continued at a frequency of once or twice per day depending on clinical response, although a few infants require more frequently than this. Ultimately in performing colonic irrigations, we aim to achieve decompression to the extent that the abdomen is no longer distended and the infant is willing to feed.

The study was registered as a service evaluation with our hospital governance team.

## Results

### General population demographics

Overall, 124 neonates presenting with distal intestinal obstruction (and with a patent anus) were identified and all were included. Of these, 86 (69%) were males. The median gestational age was thirty-nine weeks (IQR 38–40 weeks) with a median birth weight of 3330 g (IQR 3055–3660 g). Median age at presentation was 2 days (IQR 1–3 days).

Five (4%) of these neonates had bowel pathology suspected antenatally with findings of both echogenic and dilated bowel (*n* = 2), dilated bowel alone (*n* = 1), and echogenic bowel in isolation (*n* = 1). Initial symptoms and signs of the 124 neonates included vomiting at presentation in 107 (86%) which was bilious from the outset in 74, became bilious in eight and was always non-bilious in the remainder. Seventeen (14%) neonates did not present with vomiting. One hundred and eighteen (95%) neonates had abdominal distension at presentation and 93 (75%) had delayed passage of meconium. Seventy (56%) neonates presented with all three symptoms of vomiting, abdominal distension, and delayed passage of meconium. One neonate was additionally felt to have symptoms and signs of enterocolitis.

### Interventions

**Fig. 1:**
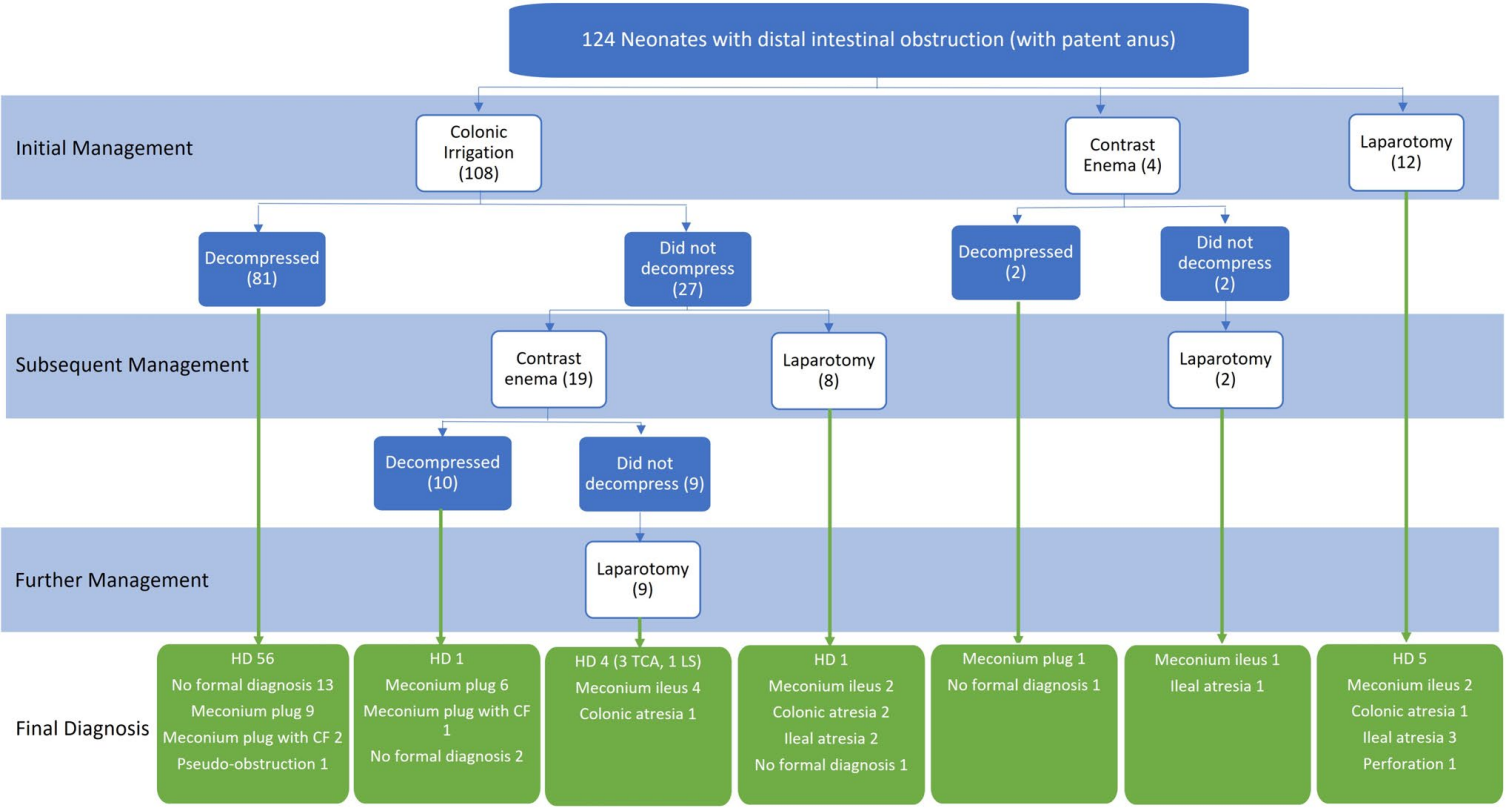
Management and final diagnosis. *HD* Hirschsprung disease; *CF* cystic fibrosis; *TCA* total colonic aganglionosis; *LS* long segment

Details of initial and subsequent interventions and final outcome are shown in Fig. 1.

The most frequent initial intervention was colonic irrigations which were performed in 108 (87%) neonates. Decompression was achieved in 81 (75%). Subsequent management in the 27 (25%) neonates in whom irrigations did not achieve decompression was contrast enema in 19 (70%) and laparotomy in 8. Of those having contrast enema after failed colonic irrigations, decompression was achieved in 37% (*n* = 10) with the remainder proceeding to laparotomy. Of those who decompressed with irrigations alone, four subsequently re-obstructed requiring surgery. All four had a final diagnosis of Hirschsprung disease although this was not known at time of surgery.

Overall, twelve (10%) neonates had an initial laparotomy. In 5 of these the indication was evidence of intestinal perforation on their abdominal radiograph, in 2 due to clinical signs of an acute abdomen and in 3 the combination of distal bowel obstruction and antenatal bowel dilatation that was suggestive of a cause requiring surgical intervention. A further 19 had a laparotomy to achieve decompression of the gastrointestinal tract following unsuccessful attempts via irrigations or contrast enema. In the 5 who underwent laparotomy on the basis of suspected perforation, findings were caecal perforation and subsequent diagnosis of Hirschsprung disease in 3, colonic atresia with perforation in 1 and terminal ileal atresia with perforation in 1.

### Additional investigations

Overall, 106 (86%) neonates had SRB taken during their initial admission. Sixty-six (62%) of these resulted in a diagnosis of Hirschsprung disease.

All but two of the 81 neonates who achieved decompression with irrigations alone had SRB. One neonate was diagnosed with meconium plug and cystic fibrosis therefore decision not to undertake SRB. The other neonate was discharged without having a SRB but was subsequently readmitted with symptoms suggestive of Hirschsprung’s enterocolitis. SRB confirmed the diagnosis of Hirschsprung disease. All of the infants who achieved decompression with contrast enema also had SRB. All but one of the neonates who passed a meconium plug had SRB, 1 did not due to family history of cystic fibrosis therefore only cystic fibrosis testing was performed which was positive.

Overall, 41 neonates were tested for cystic fibrosis, with 12 (35%) positive results. All 9 cases of meconium ileus encountered at laparotomy underwent testing for cystic fibrosis and all were positive. The majority (*n* = 15/18) of neonates who passed a meconium plug had testing for cystic fibrosis and 3 were positive. Other neonates were tested for cystic fibrosis according to surgeon preference or specific heightened suspicion. Final diagnosis in these other cases tested for cystic fibrosis was ileal atresia (*n* = 1), Hirschsprung disease (*n* = 9) no formal diagnosis (*n* = 7).

### Final diagnosis and outcome

The most common final diagnosis identified in this patient population was Hirschsprung disease affecting 67 (54%) of all neonates admitted with distal intestinal obstruction. Nine (7%) had meconium ileus associated with cystic fibrosis, and 19 (15%) had a discharge diagnosis of meconium plug syndrome (including 3 with cystic fibrosis). There were 10 (8%) neonates with intestinal atresia, (6 ileal, 4 colonic), 1 perforation without underlying aetiology identified, and 1 intestinal pseudo-obstruction. In the remaining 17 cases (14%) no formal underlying cause could be identified. This ‘no formal diagnosis’ was made as a diagnosis of exclusion with most having a SRB that was negative for Hirschsprung disease, there being no suspicion of alternate diagnosis (including some who had cystic fibrosis testing) and had adequate follow-up to be certain they were passing stool normally without concern. Just one infant was erroneously given this diagnosis initially who represented later and was found to have Hirschsprung disease.

None of this group of neonates died during the neonatal period with all surviving to discharge from the neonatal unit. Median length of neonatal stay was 11 days (IQR 7–19 days).

### Impact of antenatal diagnosis

Of the 5 neonates suspected to have intestinal pathology antenatally, three had meconium ileus, one had ileal atresia and one had meconium plug syndrome. Four had confirmed diagnosis of cystic fibrosis, one had a normal genetic screen. Four of these five neonates required a laparotomy. Thus the combination of antenatal findings and neonatal distal intestinal obstruction resulted in significant pathology in 4 of 5 cases.

### Factors associated with final diagnosis

**Fig. 2:**
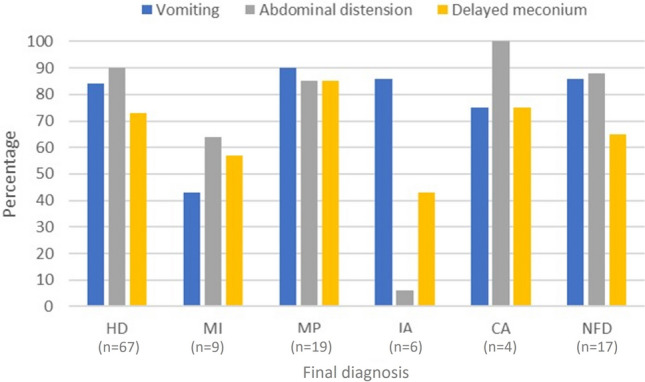
Distribution of symptoms and signs at presentation. *HD* Hirschsprung disease; *MI *meconium ileus; *MP* meconium plug syndrome; *IA* ileal atresia; *CA* colonic atresia; *NFD* no formal diagnosis

Overall distribution of key symptoms and signs at presentation was similar across all final diagnosis groups including in neonates with no underlying cause identified (Fig. 2). All three symptoms/signs—vomiting, abdominal distension and delayed passage of meconium—were present in 55% of cases of Hirschsprung disease, 36% of cases with meconium ileus, 70% of cases of meconium plug syndrome, 43% of cases of ileal atresia, 50% of cases of colonic atresia and 41% of cases with no underlying cause identified. Hence presence or absence of these clinical features was not useful as a predictor of final outcome or as a guide to interventions or investigations. Similarly there was no association between timing of presentation and final diagnosis although all these cases presented in the immediate newborn period.

In the group of neonates who could be decompressed with colonic irrigations the most common diagnosis was Hirschsprung disease (56/81; 69%) followed by no formal diagnosis (13/81; 16%). Amongst neonates undergoing other interventions, Hirschsprung disease was less prevalent (Fig. 1). Conversely most neonates ultimately diagnosed with Hirschsprung disease (56/67; 84%) could be decompressed with irrigations without requiring contrast enema. Just one was decompressed with contrast enema and the remainder required surgical decompression.

Neonates who required contrast enema to achieve decompression were most likely (10/12) to have no significant pathology (meconium plug or no formal diagnosis). All but one infant who underwent laparotomy did have significant pathology including, unsurprisingly, all 10 neonates with a final diagnosis of intestinal atresia.

All sixteen of the neonates with final diagnosis of meconium plug syndrome without cystic fibrosis achieved decompression without a laparotomy. This diagnosis was made based on passage of a meconium plug during either irrigation (*n* = 9) or contrast enema (*n* = 7, often with characteristic visualisation of meconium plugs on contrast enema) followed by no re-obstruction and negative tests for both Hirschsprung disease and cystic fibrosis (when performed). Conversely of the 20 neonates who passed a meconium plug, three had cystic fibrosis and just one had Hirschsprung disease.

Ultimately, 17 (14%) neonates had no formal diagnosis made, the majority of whom (16 [94%]) decompressed with colonic irrigation or contrast enema. One neonate who did not compress with irrigations had a persistent abnormal abdominal radiograph and proceeded to exploratory laparotomy at which no abnormality was found. Cystic fibrosis test was negative and a decision not to perform SRB was made unless the baby had further symptoms.

## Discussion

We have characterised the presenting features, interventions, investigations and ultimate diagnosis of neonates presenting with distal intestinal obstruction. In doing so, we believe we have furthered our understanding of this group of neonates and generated data that will inform clinical practice and parental counselling, and may present opportunity for quality improvement initiatives. Of note, we are unaware of any previous report that has focussed on this specific clinical presentation and subsequent outcomes. Rather most reports focus on either intestinal obstruction in general (i.e. not limited to distal intestinal obstruction) or a specific disease (e.g. Hirschsprung disease [3], meconium ileus [4]). Our approach therefore represents real-life clinical practice. Our data demonstrate that the most common ultimate diagnosis for a neonate presenting with distal intestinal obstruction is Hirschsprung disease (56%) followed by meconium plug syndrome (15%). The third most common diagnosis (14%) was that no underlying pathology was identified. Overall, just under three quarters (73%) were found to have a surgically or medically important diagnosis.

Our comparison of features at presentation (Fig. 2) failed to identify any clear patterns associated with a specific final diagnosis and similarly, we could not identify any relationship between time of presentation and final diagnosis. We acknowledge that it was not possible to determine precisely which symptom or clinical sign occurred first due to our retrospective methodology but in all cases symptoms developed in the immediate newborn period. Consequently, we believe that a standard approach to interventions in all infants presenting with distal bowel obstruction is appropriate at the outset. However, it may be possible to streamline subsequent investigations based on response to interventions. For instance, in this series successful decompression with colonic irrigations was associated with a final diagnosis of Hirschsprung disease. This finding supports SRB for investigation of possible Hirschsprung disease in all cases responding to colonic irrigations. To reinforce this, on the one occasion when SRB was not performed a case of Hirschsprung disease was missed and the child represented several weeks later with enterocolitis. Whether cases responding to colonic irrigations should all also undergo routine testing for cystic fibrosis has been frequently debated in our unit and likely by others. Our data suggest that only if a meconium plug is passed at decompression (by either irrigation alone or contrast enema) then testing for cystic fibrosis would also be appropriate; 3 of 19 cases of meconium plug syndrome had cystic fibrosis. This is similar to a previous recommendation for neonates who decompress with passage of a meconium plug [5]. We also found (although we acknowledge that the numbers are small) that amongst neonates presenting with distal intestinal obstruction in whom there has been an antenatal suspicion of intestinal pathology there was a high chance (80%) of surgical pathology requiring laparotomy. Therefore, a low threshold for laparotomy would be recommended in this group.

Comparison of our cohort to others is challenging due to the paucity of similar cohorts amongst the literature. When compared to a population of infants who were ultimately diagnosed with Hirschsprung disease, we encountered a higher incidence of infants with the ‘classic triad’ of all 3 presenting symptoms/signs of distal intestinal obstruction (abdominal distension, vomiting, delayed passage of meconium)—58% in our series compared to 26% in a population-based report of Hirschsprung disease [6]. This likely reflects our focus on the neonatal period as well as on a broader range of neonates. We believe our report is likely representative of pathology and practice in the United Kingdom since a similar proportion of neonates with Hirschsprung disease achieved decompression with colonic irrigations in our series when compared to a national population-based cohort [7].

When compared to a group of infants with meconium ileus reported in a population-based cohort, we note that no infant with meconium ileus in our series achieved decompression without a laparotomy compared to 36% in a larger series [4]. Additionally, all our cases of meconium ileus in our series were found to have cystic fibrosis. This likely represents small numbers and sampling variation rather than any fundamental differences.

The group of babies with no formal diagnosis identified and those who had a diagnosis of meconium plug syndrome not associated with cystic fibrosis are a particularly interesting group. It is challenging to explain why they have developed neonatal intestinal obstruction. Furthermore, since they typically have no long-term sequelae, it could be argued they would be suitable for a more streamlined series of interventions if it could be achieved safely. Sadly, our data provide little insight into aetiology. It is possible that there are maternal factors that may contribute to this neonatal presentation and both maternal diabetes and maternal medication use have been postulated. Unfortunately, we were not able to reliably interrogate maternity records for this study so unable to report on any such associations. It is also possible that some of this group may have had what others have termed hypoplastic left colon syndrome, a clinical entity associated with maternal diabetes and typically diagnosed on contrast enema. Our low use of contrast enema, particularly as a diagnostic tool may mean that we have overlooked this diagnosis, yet we do not believe there is any clinical significance to this since these infants have no ongoing intestinal problems.

A key difference to our management and investigation strategy compared to other units that we recognise is that we perform more colonic irrigations and very few contrast enemas in this population (either for therapeutic or diagnostic purposes) compared to other institutions [3, 6]. Whilst we acknowledge that others have reported useful diagnostic information from contrast enema that may guide future management and investigations [8], this is not universal [9] and has not been our experience. Overall, we have been less convinced of the diagnostic value of contrast enemas and as our data demonstrate, we can achieve good results with colonic irrigations as our primary treatment strategy. As such, we suggest that contrast enema is an unnecessary first step in the evaluation of neonates presenting with distal intestinal obstruction but can be reserved as a therapeutic intervention with potentially diagnostic benefit in those in whom colonic irrigation is not decompressive. This strategy has the benefits of obviating the radiation burden, inconvenience, and cost of contrast enemas. We found a median length of neonatal unit stay of 11 days. Although we have not reported absolute numbers of each type of intervention or investigation, the burden of care for these babies and their families is often high. During this time, this group of babies frequently receive multiple colonic irrigations, often undergo more than one SRB, and spend time establishing oral feeds. The resource use implications are significant with a long neonatal stay and interventions that must be delivered by healthcare professionals. We hope that our data may help streamline diagnostic and treatment processes in some way to reduce burden for patients, families, staff and the healthcare system.

The principal limitation to this study is its retrospective nature meaning that we were unable to identify the precise rationale for some management decisions. This is particularly relevant for the 4 cases who had initial contrast enema rather than colonic irrigations. We must assume that all cases managed with initial laparotomy had adequate indication for that treatment modality from the outset. The strengths of this report are that it includes a large number of neonates managed using a consistent approach over a 10-year period and that as intended we have focussed on a common clinical presentation rather than on a single underlying disease.

## Conclusion

In conclusion, our data support a recommendation that the initial management of neonates with distal bowel obstruction should be colonic irrigation since this is therapeutic in the majority and significantly reduces the need for contrast enema. These infants should all have SRB to investigate for Hirschsprung disease unless another diagnosis is evident and we recommend those who pass a meconium plug also undergo testing for cystic fibrosis. The evaluation and therapy of this groups of babies are multimodal and time consuming, placing burden on resources and families. We anticipate that these data may facilitate more targeted investigations and treatments to try to reduce resource use and length of stay as well as informing clinical practice and discussions with families.
